# Persistent visual impairments following mild-to-moderate ischemic stroke

**DOI:** 10.3389/fopht.2025.1505836

**Published:** 2025-05-26

**Authors:** Chamini Niroshika Wijesundera, Sheila Gillard Crewther, Tissa Wijeratne, Algis J. Vingrys

**Affiliations:** ^1^ School of Psychology & Public Health, La Trobe University, Melbourne, VIC, Australia; ^2^ Department of Neurology, Sunshine Hospital, St Albans, VIC, Australia; ^3^ Department of Medicine, Faculty of Medicine and Allied Health, University of Rajarata, Anuradhapura, Sri Lanka; ^4^ Department of Optometry & Vision Sciences, The University of Melbourne, Parkville, VIC, Australia

**Keywords:** acute ischemic stroke, eye-hand coordination, visual field, visual acuity-in-noise, vision, visuomotor function, Melbourne rapid field-neural (MRFn), UNSW Lee-Ryan Eye-Hand Coordination Test (SLURP)

## Abstract

**Background:**

Vision is rarely appraised either acutely or during recovery, following acute ischemic stroke. Our previous study found significant deficits in visual function after 2 to 3 days in ~68% of hospitalized mild-to-moderate acute ischemic stroke (AIS) patients with no comorbid eye disease. The purpose of this study was to evaluate recovery in vision after 2–6 months in a subgroup of the original participants.

**Methods:**

Visual assessments were performed within the first week of admission and 2–6 months later. Testing was achieved on an iPad and included visual acuity (VA), VA-in-noise, visual field, visual neglect, and time to complete an eye–hand coordination (EHC) task. All cases were radiologically confirmed, and 10 had left hemisphere lesions. The outcomes were compared to 20 age-matched healthy controls who were tested and retested over a similar duration using the same vision tests. The testing took 12 min.

**Results:**

During the first week of admission, 19/20 (95%) AIS patients returned normal visual acuity (>6/12 VA, *p* = 0.11), yet 11/20 (55%) had reduced VA-in-noise (*p* < 0.000).Visual neglect was present in 2/20 cases. Visual field defects were present in 16/20 (80%, *p* < 0.001), with 7/16 (44%) being unaware of their visual field loss. All of the patients chose to use their dominant right hand despite 10 having left hemisphere lesions and 13/20 (65%, *p* < 0.001) returning longer times to complete the EHC tracing tasks. After 2–6 months of recovery, all stroke patients returned normal visual attention, although 3/20 (15%) continued to show reduced VA in the presence of noise masks. Seven out of 20 (35%) retained visual field defects, and 8/20 (40%, three right and five left hemisphere lesions) had visuomotor impairment. Posterior circulation territory strokes and left hemisphere lesions were more likely to result in a persistent visual field loss and visuomotor deficit.

**Conclusion:**

Given that stroke is the leading cause of neurological disability affecting over 110 million people, our findings highlight the necessity for both acute and longitudinal vision assessments subsequent to mild stroke. Exposing the persistent limitations in visual functions could aid in identifying suitability for driving and the visuomotor rehabilitation of stroke survivors.

**Clinical Trial Registration:**

https://www.ANZCTR.org.au/ACTRN12618001111268.aspx, identifier ACTRN12618001111268.

## 1 Introduction

Stroke is a leading cause of neurological disability and the second leading cause of death worldwide (1–4). Yet while vision is the primary sensory information channel to the brain (5–8), driving attention (9), cognition (10), and most activities of daily living (10, 11), objective visual function is seldom routinely assessed in either acute or post-stroke settings. This is despite the seminal works of Rowe and colleagues (12, 13) in the UK, who have reported that up to 2/3 of many unselected cohorts of patients, with varying degrees of stroke severity, retain visual field loss, eye movement disorders, and perceptual deficits such as losses to contrast sensitivity for at least 12 months after the event. We have also found impaired visual acuity-in-noise, visual fields, and eye–hand coordination in greater than 68% of our previously published cohort of mild-to-moderate hospitalized acute ischemic stroke (AIS) patients who had no prior history of visual problems (14). Surprisingly, 44% of this cohort were unaware of these visual field limitations (14).

Currently, standard neurological practice in Australia typically allows stroke survivors to return to daily life and driving after 4 weeks of acute stroke recovery, provided that their neurological examination is normal based on functional assessments (15). Objective vision evaluations are limited primarily to those with observable hemianopia. Given that many of the mild-to-moderate ischemic stroke patients (14, 16) are unaware of their visual limitations, the aim of this study was to investigate recovery in visual capacity in a smaller cohort of the original group to consider the extent of recovery of visual acuity, visual fields, visual neglect, and eye–hand coordination occurring within 2–6 months of cases with mild-to-moderate stroke. We have investigated whether the change is comparable to that reported for other sensory (17) and motor functions (18–20). We expected that it is this group of mild-to-moderate stroke survivors who are likely to be discharged from the hospital without a recommendation for ophthalmic care after the acute AIS event, and they are likely to present for more regular eye care in the community.

To date, most studies (12, 21) that have performed visual evaluations in emergency departments (ED) for stroke have been restricted to visual acuity (22, 23) that is seldom impaired (14, 24) and qualitative tests (such as confrontation) that have limited sensitivity for identifying functional defects (12, 25).

The gold standard ophthalmic methodologies for assessments such as visual fields using bowl perimetry are cumbersome and expensive and require trained clinical assistants. Therefore, we have circumvented the limitations of testing by assessing visual fields, visual acuity (with and without noise masks), visual neglect, and eye–hand coordination using novel and validated (26, 27) modern technology (28). These tests are able to be presented on an iPad tablet both acutely and at retest and can be used by the patient’s bedside. Visual acuity-in-noise was included as a measure of visually driven attention and sensory processing of visual stimuli (29) following research that has indicated that noise masking is a useful biomarker for impaired visual perception in macaques with lesions in the extrastriate areas and humans with neurological conditions such as stroke (29), migraine (30), visual snow (31), and amblyopia (32).

## 2 Materials and methods

### 2.1 Participants

A total of 120 consecutive cases of acute ischemic stroke (AIS) presenting to the Sunshine Hospital Emergency Department in Melbourne between May 2018 and June 2019 were recruited for vision testing as detailed elsewhere (14). The clinical inclusion criteria required neurologist-determined, radiologically confirmed, hospitalized, first-episode acute mild-to-moderate stroke: i.e., National Institute of Health Stroke Scale >5 and <15 (13, 33–35) consistent with the proposal of Fischer et al. in 2010 (33). Mild-to-moderate AIS cases involved mild non-disabling motor symptoms at the time of the event without loss of consciousness or general attentiveness. The site of the infarction and the vascular territory affected were established and confirmed in all of our cases using brain computer tomography (CT/CT angiography) or magnetic resonance imaging (MRI/MR angiography) (34–36) as required for patient management and is reported in Wijesundera et al. in 2020 (14).

Infarcts that involved the blood supply to the posterior regions of the brain such as occipital lobe strokes and posterior cerebral artery, branches of middle cerebral, cerebellar, and basilar arteries, were defined as “posterior strokes”, whereas those involving the frontal lobe, frontoparietal regions, and the anterior branches of middle cerebral artery were defined as “anterior strokes”.

**Figure 1:**
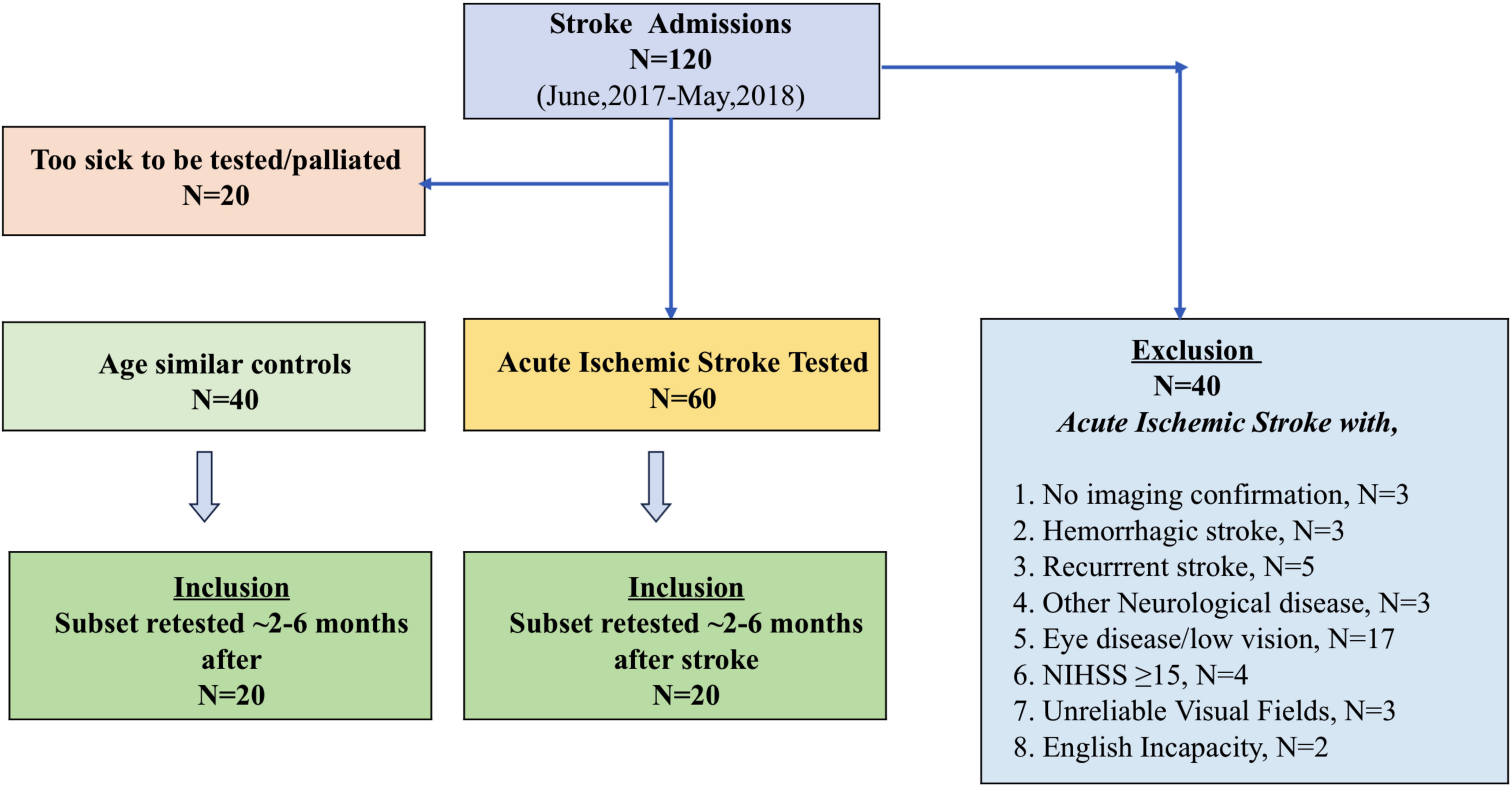
Consort Diagram: Inclusion and Exclusion Criteria for our clinical trial.

The exclusion criteria were as follows: stroke complicated by other neurological diseases, recurrent stroke, hemorrhagic stroke, clinically diagnosed ischemic stroke without radiological imaging, or ischemic stroke with NIHSS score ≥15. Two patients who had difficulty in understanding test instructions due to limited understanding of the English language and three patients who repeatedly performed unreliably (>35% false positives) on visual field assessment were also excluded (**Figure 1**). A 35% false positive rate was used to define our reliability criterion as it returns a clinically acceptable tolerance for MD error (≤1 dB from the true MD) (37).

A total of 60 “mild-to-moderate” AIS cases (age: 65 ± 14 years, male: 32/60) who had no history of eye disease were recruited and tested at their bedside following admission and diagnosis of acute ischemic stroke (days 2 to 3), and their findings have been published elsewhere (14, 37). Moreover, 45 out of 60 were classified as mild stroke (NIHSS score of ≤5). Furthermore, 20 (age: 66 ± 9 years, 12 male) of our original cohort of 60 AIS patients and 20 (age: 66 ± 7 years, 10 male) control agreed to return for retest after an average of 3.5 month (range: 2–6 months) duration. Our controls had no neurological or eye disease and were recruited following a routine optometry eye examination. We chose to recruit healthy controls who attended for clinical eye care for comparison of visual outcomes so that our data would not be confounded by other psychological (anxiety) or systemic conditions as would be expected if a hospitalized control group were used. It is this cohort of 40 participants (20 AIS and 20 controls) who are reported in the current manuscript.

All ischemic stroke patients were actively managed by the neurology team in line with hospital guidelines and specified treatments (thrombolysis/thrombectomy) for their presentation, and all patients received standard rehabilitative therapy in the period after the stroke.

### 2.2 Assessment procedure

All acute ischemic stroke patients were wearing their habitual reading glasses and had initial assessment within 5 days of admission at their hospital bedside. Testing was performed on an Apple iPad (9.7 inch) using the Melbourne Rapid Field-Neural (MRFn) (26) and UNSW (Department of Optometry, University of New South Wales, Australia) SLURP Eye–Hand Coordination applications (27). The test instructions and a practice trial required about 1 min per patient, whereas full testing of both eyes took ~12 min.

**Figure 2:**
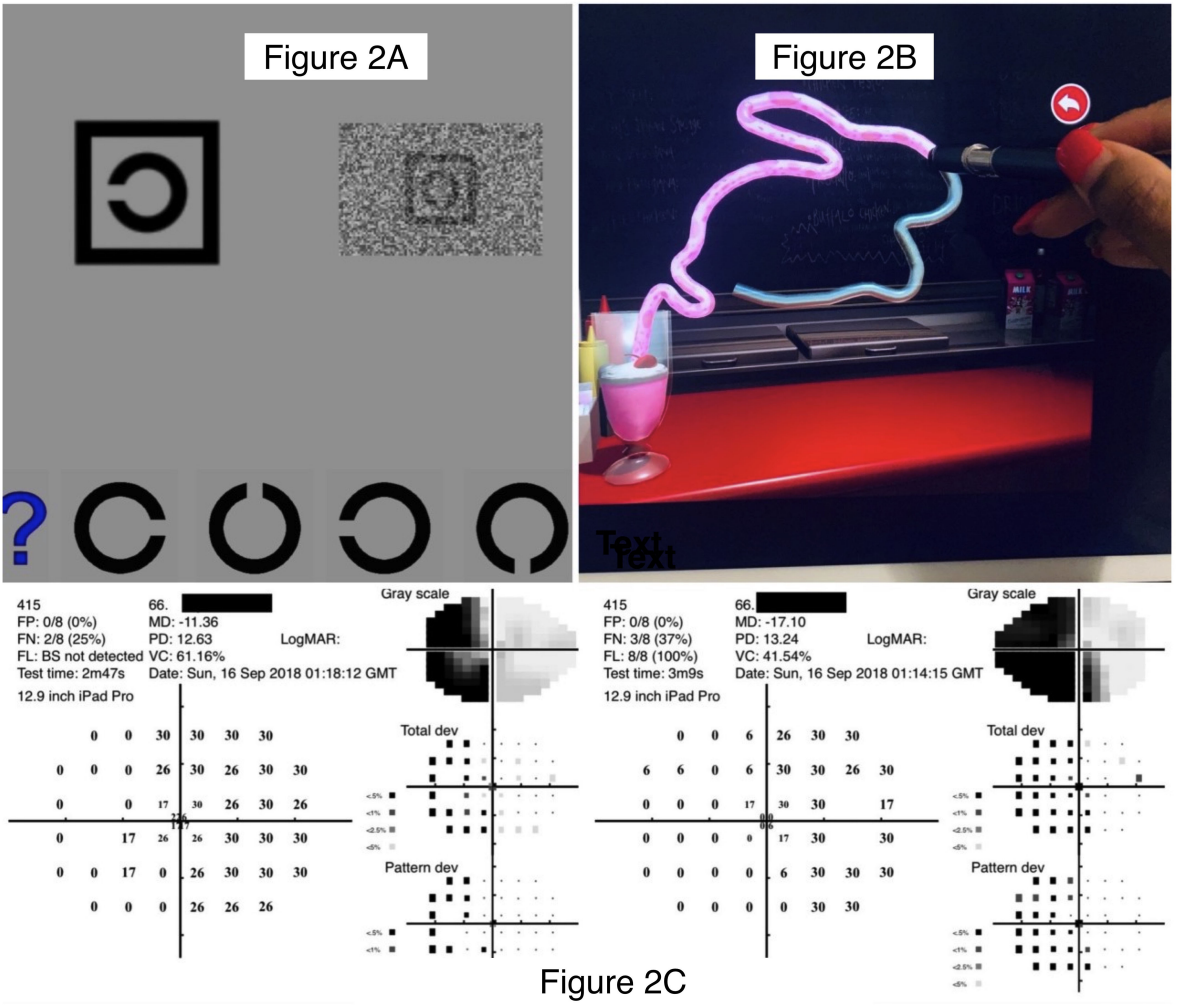
Images of the test suite used on the iPad Tablet. **(A)**: Optotype letter “C” shown with the MRFn acuity test: in high contrast (left) and dynamic noise mask (right). The participants’ task is to match the orientation of the “C” in the box at the top of the panel with the appropriate option at the bottom. High-contrast optotypes are shown first during visual acuity assessment, followed by the visual acuity-in-noise optotype. **(B)**: Top right: Tracing of the shape “Rabbit” using the SLURP Eye-hand Coordination App showing the stylus used for tracking, the completed track (pink), and the blueish white track remaining to be done. **(C)**: Outcome for a left hemianopia in a 66-year-old patient tested with the MRFn test grid which took about 3 min in both eyes. This modified grid has reduced vertical and horizontal extent (15° Å~ 21°) and four test points added near the fovea (0.8°)..

The Melbourne Rapid Fields-Neural (MRFn) application (Glance Optical Pty Ltd, https://www.visiondata.net.au) was administered monocularly at 33 cm (13 inches) starting with the right eye (RE) and high-contrast visual acuity, followed by visual acuity-in-noise (acuity-in-noise) and visual field thresholds out to ±20 degrees using a modified 24–2 pattern (**Figure 2C**) (14, 26). The EHC test was undertaken using binocular viewing after both vision tests were completed. In testing visual acuity (VA), MRFn presents single Landolt C targets shown in a box outline at one of four randomly chosen orientations. The patient’s task was to match the correct orientation (see **Figure 2A**) by screen touch or nominate the “?” symbol if unsure (see **Figure 2A**). The software identifies the lowest level (logMAR) achieved by the patient at a two of three correct criteria. We required a visual acuity of 6/12 (logMAR 0.3), being the current Australian driving license requirement, or better to indicate adequacy of reading glasses. The visual acuity-in-noise test uses the same Landolt C target shown in the presence of a dynamic luminance noise mask (**Figure 2A** shows a static image). For the visual field, a reduced and modified 24-2 (20° × 15°) test grid (see **Figure 2C**) was used to return local thresholds (in dB). These were compared to a normative database by the software to give a global mean deviation (MD, dB difference to age-similar norm). A qualitative description of the visual field loss was also determined by the researcher. The reduced 24–2 visual field test grid was chosen in our study because it does not require any fixational eye movements and is completed in 2 to 3 minutes. Patients who gave reliable visual field results (≤35% false positives) were included in our analysis; three unreliable individuals have been excluded (**Figure 1**). These vision tests were coupled with the SLURP Eye–Hand Coordination test performed binocularly (**Figure 2B**) using an iPad stylus for tracking (27, 37). The evaluation of eye–hand coordination required the participants to trace three shapes (see **Figure 2B**). If the stylus is maintained within a tolerance zone of the shape outline (± 2.5 mm) during tracing, the shape changes color (from blue to pink, **Figure 2B**), but if the participant deviates beyond the tolerance limit, the color change stops and the patient is asked to return to the location of the color border in the shape as rapidly as possible and resume tracing from the last correct location (**Figure 2B**). Detailed procedures have been reported in our previous publications (14, 37). The SLURP test was chosen for our purpose as it requires goal-directed attention and eye movements to focus on the shape and accurately plan, control, trace, and correct any eye–hand deviation errors which have been reported as having potential for change following stroke (10, 38). Our previous analysis of the number of deviations, magnitude of deviation, and completion time finds that completion time is the best index of performance (37); hence, timing is the only measure considered in the present study. Recovery was evaluated using the same tests presented in the same order at retest (~3.5 months post-stroke) following a comprehensive optometry examination. All of the patients chose to use their previously dominant right hand for tracing despite the fact that 10/20 (50%) of our first-episode stroke patients had experienced a radiologically confirmed left hemisphere lesion (39), and our data show that they do not give normal tracing performance.

Hemi-spatial neglect (HSN) was considered in all cases of stroke recovery using a line bisection test and the MRF cancellation test. The MRF cancellation test requires the patients to “tap” on 40 frowning faces (20 in each hemi-field) and change them to “smiley” faces. We consider the presence of HSN when the difference in L and R hemifield identifications exceeds 4.

Ethics approval was provided by Sunshine Hospital (Western Health Ethics Committee HREC/16/WH/1) review board, and the study was conducted in accordance with the tenets of the Declaration of Helsinki with all of the participants (or their carers) giving signed informed consent to participate. Our study was registered as a clinical trial at ACTRN12618001111268.

## 3 Data analysis

Non-parametric statistical tests were used to compare the stroke and control groups given the non-gaussian data distributions. Kruskal–Wallis tests were used to identify differences between group medians, with Dunn’s multiple comparison applied to identify significant differences between groups. Although each eye was tested, the eye ipsilateral to the CT/MRI defined lesion was analyzed in the stroke group as each eye is driven by 5/6 ipsilateral eye muscles, and hence the visual function of the eye ipsilateral to the infarct is more likely to be affected in the AIS than the contralateral eye (40). We compared this eye to the RE of controls as the visual acuity of the two eyes of the controls was similar. Analysis of the fellow eye in our data set does (stroke or normal groups) not alter the general findings.

The performance of our worst control (one of 20) was used to define a non-parametric 95th percentile to define “abnormal” outcomes. Group performance is specified with 95% confidence intervals. Statistical analysis was conducted using GraphPad Prism v7.00 for Windows (www.graphpad.com), and a *p*-value of 0.05 was used to indicate statistical significance in our results.

## 4 Results

### 4.1 Vision deficits in the acute phase of ischemic stroke

Normal visual acuity (>6/12) was the consistent finding in 19 of our 20 (95%) AIS patients in the acute phase, although around half (11/20, 55%) of the participants demonstrated significant deficits for visual acuity-in-noise, visual field loss (16/20, 80%) and had a prolonged tracing time on the eye–hand coordination task (13/20, 65%) compared to controls. The acute visual field losses involved 9/16 patients with hemianopia, 4/16 quadrantanopias, and 3/16 altitudinal defects. Despite these limitations to vision and visuomotor capacity, 7/16 (44%) patients with these severe visual field defects were unaware of their visual limitation.

### 4.2 Recovery in visual deficits

### 4.3 Visual acuity

**Figure 3:**
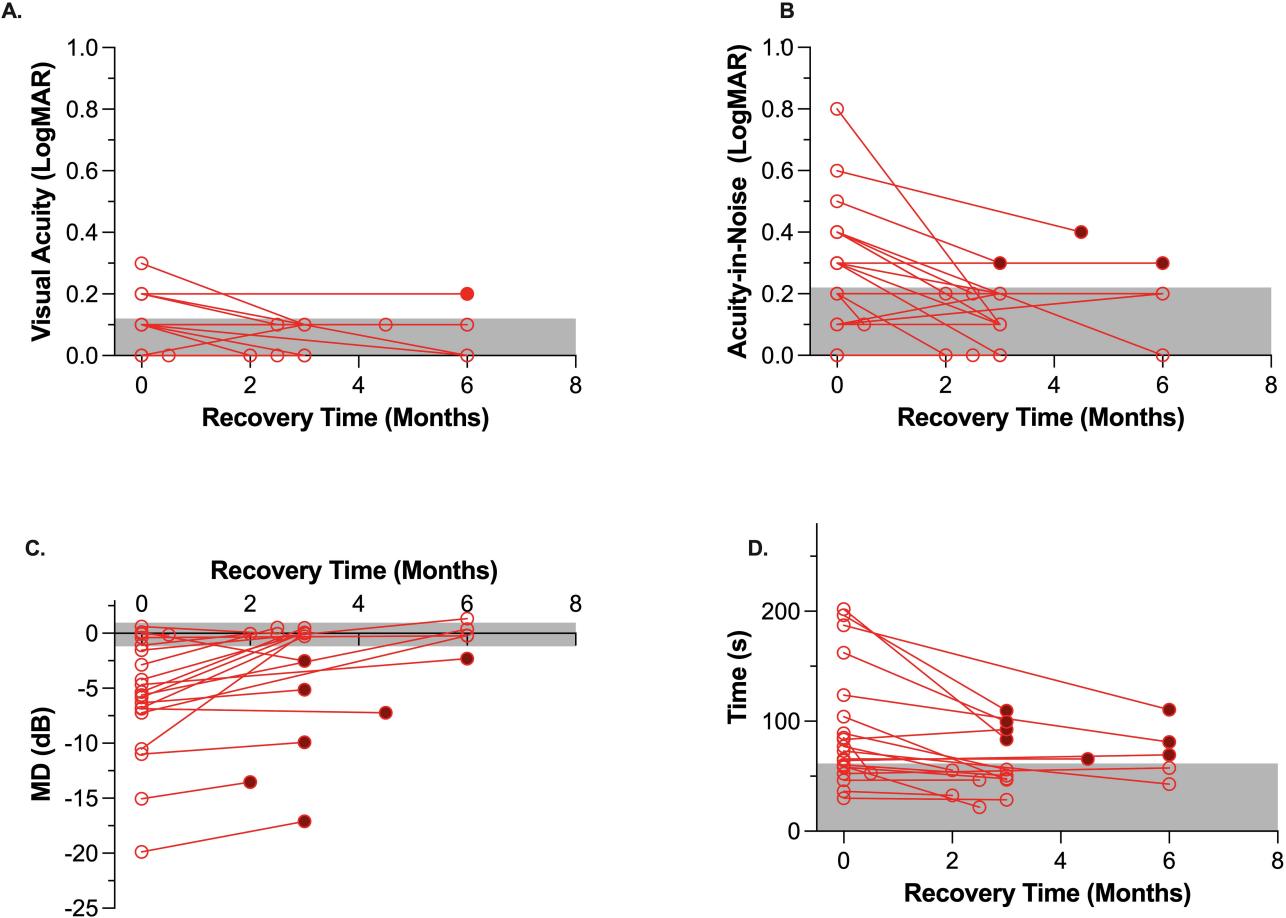
Change in visual function of the stroke cohort (n = 20) between acute assessment in hospital (i.e., unfilled circles on left) and at the exact retest time shown on the x-axis. Filled symbols at retest time indicate cases which remained beyond the worst control (gray area). The gray zone indicates the 95% confidence interval for age-similar controls. **(A)**: Visual Acuity, **(B)**: Visual acuity-in-noise, **(C)**: Visual Field Mean Deviation (MD-dB), 3D: Eye-hand coordination time (s).

**Figure 4:**
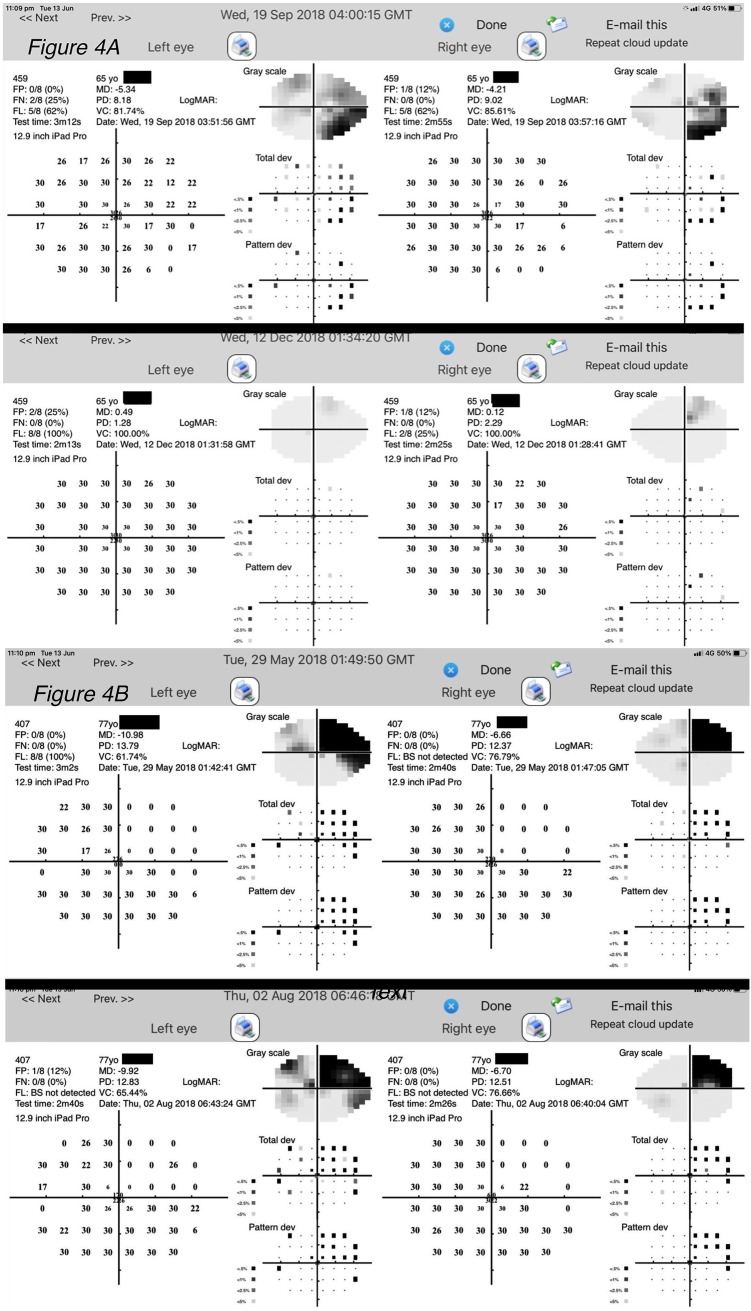
**(A)** An example of a 65-year-old stroke patient with a right inferior quadrantanopia that resolved by the time of retest. Top: Visual field measured during the acute phase of stroke on admission to the hospital (September 19, 2018). Bottom: Visual field measured at retest (December 12, 2018). **(B)** Stable visual fields measured from a patient showing a right superior quadrantic defect. This patient shows no change in their visual field 2.5 months after stroke as evident qualitatively and in the MD values. Top: Visual field measured during the acute phase of stroke on admission to the hospital; MD LE -10.98 and RE -6.66. Bottom: Visual fields measured at 2.5 months later return MD of LE -9.92 and RE -6.70.

Only one patient with a large right posterior cerebellar artery stroke had abnormal visual acuity (6/12) at the initial assessment. This patient returned near normal visual acuity of 6/7.5 after 3 months of recovery. On the other hand, 10 of the 11 of our AIS group who initially had abnormal (<6/12) visual acuity-in-noise showed a significant improvement in their visual acuity-in-noise at retest (mean, test: 6/12, retest: 6/7.5, *p* < 0.001) with eight of the 11 (72%) returning normal outcomes for this test (**Figures 3**, **4**). The three patients who continued to show abnormal visual acuity-in-noise at retest involved a right frontal lobe and two occipital lobe lesions. No substantial changes were found in controls for visual acuity-in-noise at retest (controls: mean, test: 6/7.5; retest: 6/6, *p* = 0.60).

### 4.4 Visual fields

**Figure 5:**
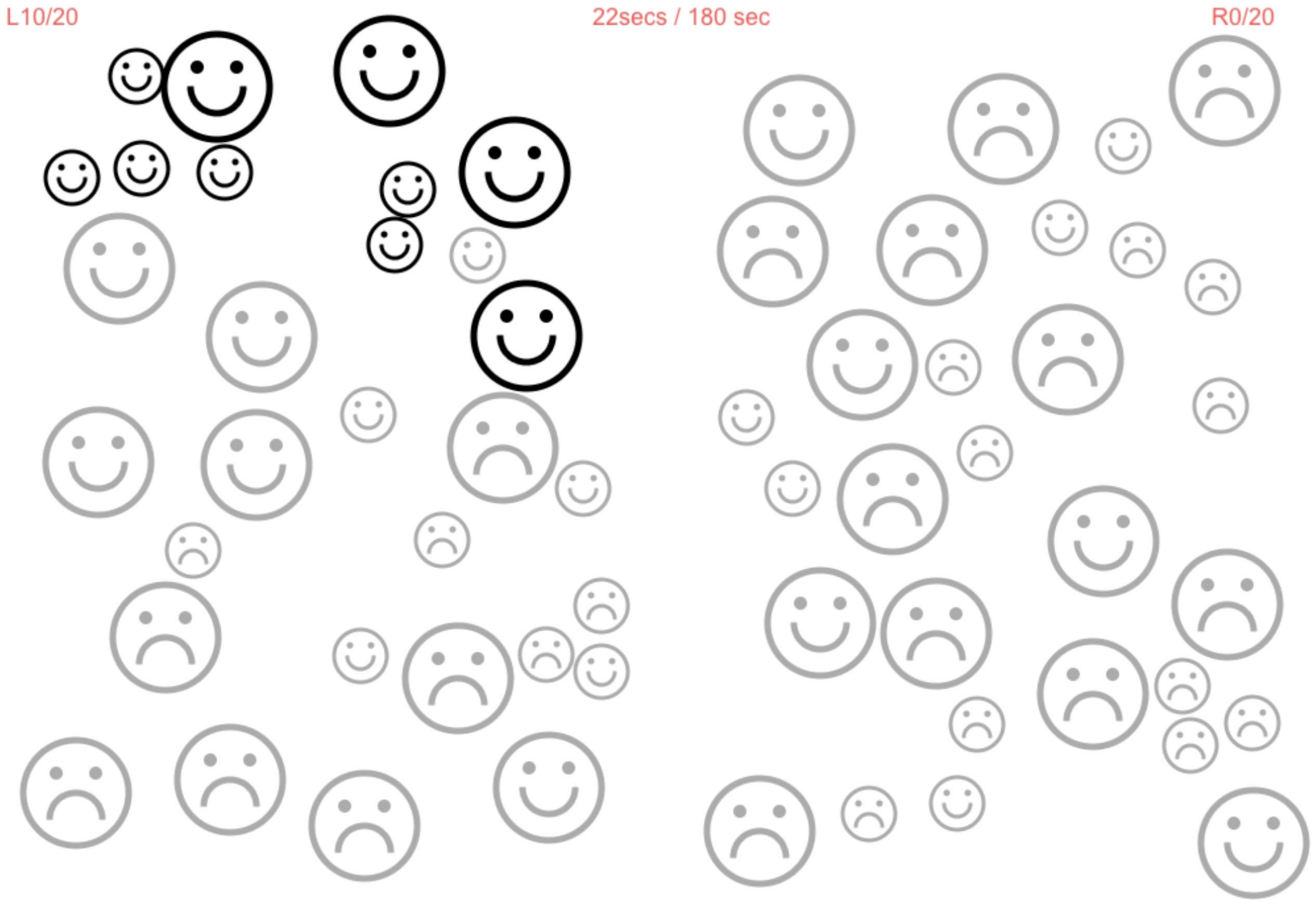
MRF hemispatial neglect cancellation test.

Our recovery analyses (**Figure 3**) indicates that visual field thresholds for mild-to-moderate stroke patients can improve in both the global MD index (AIS: -5.7 ± 5.5 dB, retest: -2.8 ± 5.4 dB, *p* < 0.001) and in the manifest form of visual field scotoma after an average recovery time of 3.5 months (**Figures 3A**, **4**, **5C**). Indeed of the 16 AIS participants who initially showed abnormal visual fields, 9/16 (56%) showed a substantial improvement at retest (**Figure 3**), with normalization of their initial quadrantanopic, hemianopic, or altitudinal visual field loss, whereas 4/16 (25%) had partial recovery and 3/16 (38%) remained unchanged and abnormal at retest. Overall, 7/20 (35%) stroke participants showed significant and persistent visual field defects at retest.

We found that two of our cases (10%) had parietal stroke with visual neglect. They failed the line bisection test and the MRF cancellation test (**Figure 5**) contralateral to the site of lesion. Both patients correctly identified all 20 faces on the side of lesion, but only 13/20 on the contralateral side. The visual field losses found acutely in these patients recovered in both cases.

### 4.5 Visuomotor function: eye–hand coordination

Acutely, 13 out of the 20 (65%) stroke patients returned significantly slower eye–hand tracing times. Eight of 13 (62%) had left hemisphere lesions, and five (38%) had right hemisphere lesions, but all chose to use the iPad stylus in their previously dominant right hand. At retest, eight of the 13 had maintained this visuomotor defect, whereas the other five (38%) showed significant recovery and normal eye–hand coordination tracing time (**Figure 3**). The lesions of the eight individuals whose visuomotor function did not improve were posterior infarcts, with 5/8 experiencing left hemisphere (**Figure 4**) lesions.

### 4.6 Recovery of visual deficits post-stroke in relation to brain blood supply

**Figure 6:**
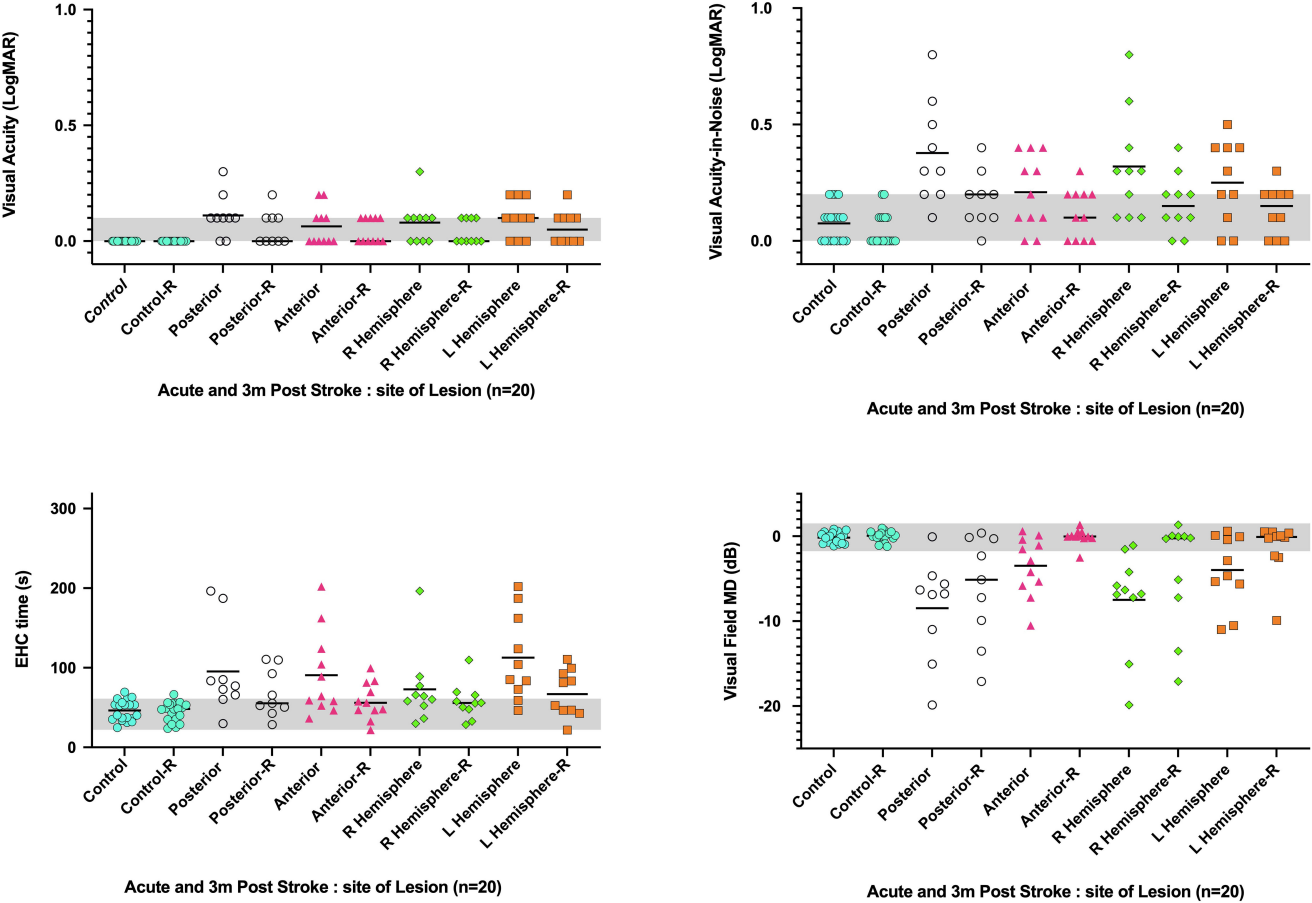
Visual abnormalities observed acutely and after 3.5 months average recovery time (-R) in relation to the site of stroke lesion (i.e., right or left hemisphere and anterior or posterior blood supply). Y axis: visual function parameter, X axis: Acute and recovery site of lesion. The bar identifies the cohort mean.

Although many patients initially showed significant impairment in visual acuity-in-noise, these cases did not show any particular association with anatomical location of the lesion or blood supply (**Figure 4**). On the other hand, in the 16/20 AIS lesions that initially resulted in visual field defects, posterior right-sided lesions give significantly worse outcomes compared with anterior or left-sided lesions (**Figure 6**). A significant increase in eye–hand coordination tracing time is likewise associated with left-sided lesions.

### 4.7 Nature of vision loss

**Figure 7:**
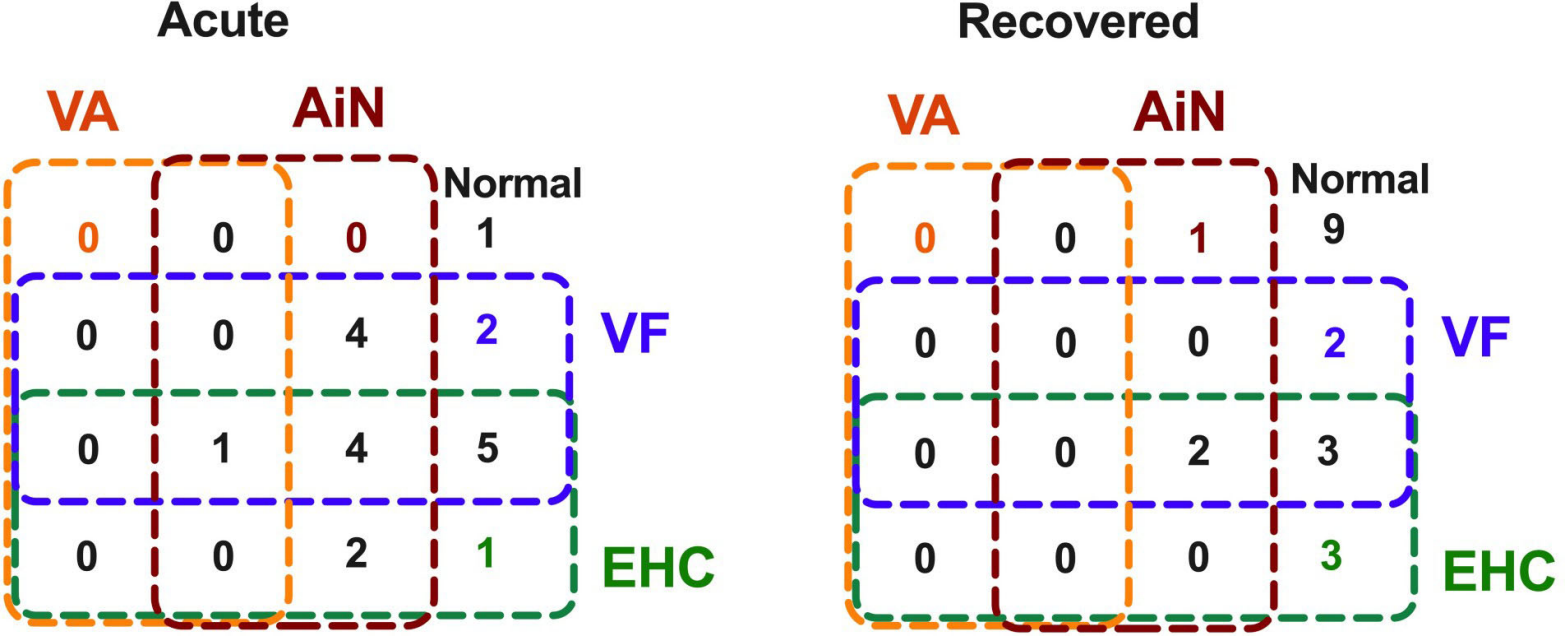
Venn diagram: Association of vision changes at acute onset and on recovery in 20 cases of mild-to-moderate acute ischemic stroke.

As shown in the Venn analysis, **Figure 4** summarizes impaired visual functions assessed during the acute and recovery phases. Notably, 19/20 (95%) of our radiologically confirmed AIS cases showed (**Figure 7**, left) at least one aspect of impaired vision immediately on presentation for stroke. The fact that 4/20 (20%) show impairment in all three key aspects of vision, i.e., visual acuity-in-noise, visual field thresholds, and eye–hand coordination tracing time, indicates that each aspect of this test battery is identifying unique deficits associated by the stroke. Significant improvement was found in all aspects of vision after 2–6 months, with 9/20 (45%) returning normal visual capacity and none showing impairment in all aspects of vision (**Figure 7**, right). Persistent visual acuity-in-noise deficits were present in 3/20 (15%) cases and visual field loss in 7/20 (35%), and 8/20 (40%) retained eye–hand coordination delays.

## 5 Discussion

The aim of this study was to explore the persistence of abnormal vision following mild-to-moderate ischemic stroke. A subset of 20 of the original cohort of 60 radiologically confirmed mild-to-moderate AIS patients (14, 37), of whom 44% of the original cohort were unaware of their visual field deficits (14, 37) were recruited to this trial as mild-to-moderate stroke survivors. The average age of our sample was 66 ± 8 years, who are likely to be mobile, driving, and visiting ophthalmic practices and already receiving age-related vision management. Hence, they are likely to benefit from comprehensive functional vision assessment following recovery from stroke and to consider any limitations to mobility and daily activities.

Our most noteworthy finding is that only half of the stroke cases recovered normal visual function over the 3- to 4-month period despite most having normal central visual acuity immediately after the acute cerebrovascular event. Sparing of central VA was expected given the dual foveal projection that projects to both hemispheres, resulting in macular sparing (41–43). We adopted visual acuity measurement in our trial as VA is often needed in diagnosing acute neuro-ophthalmologic presentations. Furthermore, previous clinical trials on acute stroke which included patients with vision problems and more severe expression of stroke found the visual acuity to be reduced in up to 56% (23, 44, 45) of the cohort. In our trial, we excluded 16 stroke patients with a past record of eye disease from our test cohort. The *post-hoc* analysis of all of our data (including these 16 cases) finds that having poor visual acuity (≤6/12) is a good indicator of the presence of co-morbid eye disease in a stroke patient, yielding a likelihood ratio of 5.7 for ocular co-morbidity. Our data find that a loss of VA does not often occur in mild stroke. Indeed previous trials reported by Rowe et al. (12, 44, 46) suggest that reduced visual acuity can be associated with severe expressions of stroke.

Visuomotor capacity and visual field thresholds show the least recovery, leaving nearly half of the stroke survivors with induced visual defects (**Figure 7**) and 25% (5/20) of them with concomitant visuomotor and visual field defects.

Abnormal visual fields (mean deviation) were found in 16/20 (80%) of our test cohort during the acute phase. Residual visual field defects remained in 7/20 (35%) at retest. Six out of the seven stroke patients whose visual fields did not normalize included four occipital lobe and two posterior cerebellar artery infarcts with contralateral hemianopic and quadranatopic defects showing partial improvement in the form of developing incongruous hemianopias (47). Notably, all four occipital lobe patients showed no improvement in their hemianopia or quadrantanopia at retest, meaning that in terms of recovery, our findings are similar to those previously reported in the literature (48) where 39% of the patients show partial recovery of visual field defects, 7.5% showed full recovery, and 52% retain the loss in visual field sensitivity. Our observations suggest that acutely impaired visual function following stroke is a more generalized response to vascular dysfunction than infarct location *per se*. Many cases can recover from this acute event, but those who do not may fail to do so because their lesion-specific locations affect their vision as reported by Hayes and Merigan in macaques and humans a long time after their stroke (29).

A consistent improvement of ≥1 dB in mean deviation on successive visual field tests (L, R, L, R) has been suggested as due to a “learning effect” (49). We investigated our data for such trends and find that three of our 20 acute ischemic stroke test/retest patients (15%) met this criterion, indicating that this potential confound arises from learning rather than recovery in cortical function and needs to be considered by clinicians in evaluating stroke cases especially in considering recovery.

Significantly increased eye–hand coordination time was initially required by 13/20 (65%) of our cohort, with only 5/13 (38%) showing recovery. Of the 8/20 of the retest cases who continued to show abnormal EHC, five had left-sided lesions, suggesting that the increase in visuomotor “timing” (37) is likely related to the patient’s impaired visually driven motor capacity.All of our results confirm the large body of data previously published by Rowe and colleagues (12, 44) in the UK, highlighting the fact that AIS contributes significantly to many persistent impairments in vision, eye movement dysfunction, and visual field defects (50, 51) and probably also to binocular dysfunction (52) and mis-coordination between ocular and motor function and have a negative impact on the quality of life. Quality of life is also likely to be further compromised by persistent visual deficiencies that result in an increased need for additional conscious visual attention to simple visuomotor activities [such as lifting a hand or walking (53, 54) or reading, watching television, holding a cup of water, grasping for an object, or driving (55, 56)]. The need for such conscious additional attention has previously been reported to induce increased fatigue (57), concentration, higher risk of falls, and anxiety and negatively affect mood (58). Importantly, for the 7/20 (35%) persistent quadrantanopic or hemianopic defects, it would also be expected to impact negatively on their mobility and capacity to drive as well as reading and tracking. It is vital for eye care practitioners to understand the impact of left hemianopias causing trouble in picking up the start of a line when reading versus right hemianopias causing asthenopia when reading from left to right and to implement thorough binocular vision assessment as per the patient’s daily activities.

Impaired visual acuity-in-noise that is usually associated with impaired visual attention (59) in other neurological conditions (30, 32, 60) such as migraine (30), amblyopia (32), schizophrenia (60), and autism spectrum disorder (61) has not previously received much consideration either acutely nor early in recovery in stroke (29). Indeed in our retest subset, 11/20 (55%) were acutely affected, and eight improved over time, with deficits only persisting in 3/20 (15%) patients 2–6 months later. Two out of the three patients with persistent visual acuity-in-noise deficits at retest had occipital lobe lesions, whereas the third had a right frontal lobe lesion and returned 6/12 visual acuity-in-noise at both test and retest, implying that visual processing and the ability of vision to drive conscious attention, memory, and visuomotor areas (41, 62) remain impaired if the parieto-frontal network (63) is permanently damaged (9, 64, 65). Our findings highlight the importance of acute visual assessment following stroke in patients, particularly in those with posterior stroke who are likely to benefit from early visual rehabilitation to deal with edema-induced impairments in visual attention and contrast sensitivity that are likely to affect mobility and daily life.

## 6 Limitations

Although the generalizability of this study could be expected to be limited by the size of the sample of mild-to-moderate stroke patients without eye disease and in terms of limited diversity in age, ethnicity, and comorbid conditions in a hospitalized environment that is usually associated with severe strokes, the similarities of our retest results to previous less stringently selected patient samples where such cases were included (12, 44, 46) increase its likely importance as an early biomarker of visual dysfunction immediately following stroke as well as on recovery and indicate the need for incorporation of such visual assessment into stroke rehabilitation programs. More constraining to long-term understanding of the temporal trajectory of AIS pathophysiology for our work is having only one time point of short-term recovery, namely, 3 months, that limits understanding of the duration of time needed for recovery of different visual functions. It is also possible that early treatment with thrombolysis in ischemic stroke patients may have produced a lessened functional impact than has previously been reported following AIS (66, 67).

## 7 Conclusion

Our findings demonstrate the presence of acute and persistent vision deficits after 2–6 months in ~50% of mild-to-moderate AIS survivors of whom 44% were initially unaware of their visual deficits. Our results highlight the consequences on visual function of a mild AIS event and the need for comprehensive regular monocular and binocular visual examinations and the significant implications for each patient’s recovery and rehabilitation as undiagnosed visual impairments may hinder full functional recovery and quality of life.

Our Venn diagram also highlights that 95% of acute AIS cases can be correctly identified using three vision tests (acuity-in-noise, visual field, and EHC) in 10 min of testing. This information should be useful for first responders in the early diagnosis of stroke and later for eye care providers diagnosing and managing stroke and post-stroke patients.

## Data Availability

The original contributions presented in the study are included in the article/**Supplementary Material**. Further inquiries can be directed to the corresponding author.
